# Pneumococcal Carriage in Burkina Faso After 13-Valent Pneumococcal Conjugate Vaccine Introduction: Results From 2 Cross-sectional Population-Based Surveys

**DOI:** 10.1093/infdis/jiab037

**Published:** 2021-09-01

**Authors:** Lassané Kaboré, Tolulope Adebanjo, Berthe Marie Njanpop-Lafourcade, Soumeya Ouangraoua, Felix T Tarbangdo, Bertrand Meda, Srinivasan Velusamy, Brice Bicaba, Flavien Aké, Lesley McGee, Seydou Yaro, Edouard Betsem, Alain Gervaix, Bradford D Gessner, Cynthia G Whitney, Jennifer C Moïsi, Chris A Van Beneden

**Affiliations:** 1 Agence de Médecine Préventive, Ouagadougou, Burkina Faso; 2 Institute of Global Health, University of Geneva, Geneva, Switzerland; 3 National Center for Immunization and Respiratory Diseases, Centers for Disease Control and Prevention, Atlanta, Georgia, United States of America; 4 Centre Muraz, Bobo-Dioulasso, Burkina Faso; 5 Davycas International, Ouagadougou, Burkina Faso; 6 Ministère de la Santé, Ouagadougou, Burkina Faso; 7 Pfizer, Paris, France; 8 Faculty of Medicine and Biomedical Sciences, University of Yaoundé 1, Yaoundé, Cameroon; 9 Department of Pediatrics, University Hospitals of Geneva, Geneva, Switzerland; 10 Agence de Médecine Préventive, Paris, France; 11 Pfizer, Collegeville, Pennsylvania, USA

**Keywords:** *Streptococcus pneumoniae*, survey, carriage, serotypes, pneumococcal conjugate vaccine, Burkina Faso

## Abstract

**Background:**

Burkina Faso, a country in Africa’s meningitis belt, introduced 13-valent pneumococcal conjugate vaccine (PCV13) in October 2013, with 3 primary doses given at 8, 12 and 16 weeks of age. To assess whether the new PCV13 program controlled pneumococcal carriage, we evaluated overall and serotype-specific colonization among children and adults during the first 3 years after introduction.

**Methods:**

We conducted 2 population-based, cross-sectional, age-stratified surveys in 2015 and 2017 in the city of Bobo-Dioulasso. We used standardized questionnaires to collect sociodemographic, epidemiologic, and vaccination data. Consenting eligible participants provided nasopharyngeal (all ages) and oropharyngeal (≥5 years only) swab specimens. Swab specimens were plated onto blood agar either directly (2015) or after broth enrichment (2017). Pneumococci were serotyped by conventional multiplex polymerase chain reaction. We assessed vaccine effect by comparing the proportion of vaccine-type (VT) carriage among colonized individuals from a published baseline survey (2008) with each post-PCV survey.

**Results:**

We recruited 992 (2015) and 1005 (2017) participants. Among children aged <5 years, 42.8% (2015) and 74.0% (2017) received ≥2 PCV13 doses. Among pneumococcal carriers aged <1 year, VT carriage declined from 55.8% in 2008 to 36.9% in 2017 (difference, 18.9%; 95% confidence interval, 1.9%–35.9%; *P* = .03); among carriers aged 1–4 years, VT carriage declined from 55.3% to 31.8% (difference, 23.5%; 6.8%–40.2%; *P* = .004); and among participants aged ≥5 years, no significant change was observed.

**Conclusion:**

Within 3 years of PCV13 implementation in Burkina Faso, we documented substantial reductions in the percentage of pneumococcal carriers with a VT among children aged <5 years, but not among persons aged ≥5 years. More time, a change in the PCV13 schedule, or both, may be needed to better control pneumococcal carriage in this setting.

*Streptococcus pneumoniae* (pneumococcus) is a major cause of disease and death globally [1, 2]. Persons who acquire pneumococci in their upper respiratory tract may develop clinical disease or, more likely, become asymptomatic carriers [3]. Healthy pneumococcal carriers are responsible for most transmission of pneumococci between persons. Therefore, carriage plays a critical role in the epidemiology of pneumococcal disease. Pneumococcal conjugate vaccines (PCVs) prevent carriage of vaccine serotypes and have proven very effective in reducing the burden of pneumococcal disease, even among unvaccinated contacts of vaccine recipients–so called indirect effects or “herd immunity” [4–6].

The 13-valent PCV (PCV13) was introduced into Burkina Faso in October 2013 with 3 primary doses given to infants at age 8, 12, and 16 weeks without a booster (“3 + 0” schedule) or a catch-up campaign [7]. Postintroduction studies are needed to fully understand the effects of the vaccine and provide scientific evidence to decision makers on whether adjustments in the PCV program are needed to better control pneumococcal disease. An analysis of national bacterial meningitis surveillance data collected in the first years after PCV13 introduction suggested that the PCV13 program reduced the pneumococcal meningitis burden in the country [8], but vaccine effects on carriage have not yet been reported.

Before PCV13 introduction (2008), a pneumococcal carriage survey was conducted in the city of Bobo-Dioulasso [9], providing useful baseline data to monitor vaccine effectiveness. Subsequently, we conducted 2 cross-sectional population-based surveys of pneumococcal carriage in the same city, approximately 1 year (March 2015) and 3 years (March 2017) after PCV13 introduction. We summarize data from these 2 studies, and through comparisons with published pre-PCV carriage data from 2008, estimate the impact of PCV13 against vaccine-type (VT) pneumococcal carriage in the region.

## METHODS

### Study Design and Participants

Each study (2015 and 2017) was a population-based cross-sectional survey conducted in the city of Bobo-Dioulasso, located in western Burkina Faso. We aimed to recruit 1000 participants per survey, with 200 participants in each of the following age groups: 0–11 months (1–11 months in 2017), 12–23 months, 24–59 months, 5–14 years and ≥15 years.

Similar to the 2008 survey, the 2015 and 2017 surveys used an age-stratified cluster sampling method to recruit participants. Ten urban districts (of the 21 that existed at the time of the 2008 survey) were randomly selected [9]. For each of these districts, 20 crossroads were randomly identified to serve as starting points. From each starting point, a street was randomly selected, and all the compounds along that street were visited by field workers, starting from the first compound on the left-hand side of the street. 

A representative of each of the 5 age groups was recruited in each compound. If there were >2 representatives for the same age group, 1 was selected at random. If an age group was not represented in a given compound, field workers proceeded to the next compound in search of eligible subjects. Participants could be from the same family or not, depending on the composition of the household (we did not track clustering by family). Eligible subjects who consented to participate were screened by a questionnaire and then referred to the clinic of the Centre Muraz (https://www.centre-muraz.bf) for collection of nasopharyngeal (NP) (all ages) and oropharyngeal (OP) swabs (participants ≥5 years of age only).

### Data Collection

After informed consent was obtained, a standardized questionnaire was administered to the parent or guardian of the participating child or to the participating adult. Questions included basic demographics, vaccine history, household characteristics (eg, number of persons living in the household, types of fuel used for cooking, and exposure to smoke), and socioeconomic variables. Children’s vaccination histories were obtained from vaccination cards and included data on receipt of PCV13 and other routine immunizations. To obtain documented immunization histories, the study team reviewed immunization registers in health facilities for children whose vaccination cards were unavailable or failed to provide vaccination dates. During the visit for collection of NP and/or OP specimens, a second questionnaire was administered that included questions about current respiratory symptoms, recent illnesses, history of meningitis or pneumonia, and recent use of antibiotics.

### Specimen Collection and Management

At the study clinic, NP and OP swab samples were collected by trained staff, following World Health Organization consensus methods [10]. OP swab samples were processed separately and collected in addition to NP swab samples to increase the efficiency of pneumococcal isolation. Samples were immediately placed into skim milk, tryptone, glucose, and glycerin (STGG) transport medium in appropriately labeled cryovials and placed on an icepack. On reaching the laboratory, inoculated STGG medium was vortexed for 10–20 seconds to disperse organisms from the swab sample. The samples were then either plated immediately (2015) or frozen at −80°C and processed later (2017).

### Laboratory Procedures

For samples from 2015, 10 µL of STGG medium was directly streaked on a sheep blood agar plate containing 7% gentamicin. Samples from 2017 were first enriched by transferring 200 µL of STGG medium to 5.0 mL of Todd-Hewitt broth containing 0.5% yeast extract and 1 mL of rabbit serum. After incubation at 35°C–37°C for 6 hours, 10 µL of cultured broth was plated on sheep blood agar. Plates were incubated overnight at 37°C in a 5% carbon dioxide atmosphere. After 18–24 hours of incubation, pneumococci were identified by catalase, optochin susceptibility and bile solubility testing. 

Isolates from the 2015 study were also confirmed using *lyt*A polymerase chain reaction (PCR) [11]. All *S. pneumoniae* isolates were stored in STGG medium at −80°C. To increase the sensitivity of pneumococcal detection*, lyt*A PCR testing was also performed on all STGG specimens collected in 2015 that were negative by culture; specimens that tested *lyt*A positive underwent repeated culture, and any pneumococci isolated were serotyped. Pneumococcal serotypes were determined for cultured isolates by a sequential multiplex conventional PCR assay [12]. All pneumococcal isolates determined to be nontypeable or for which the serotype was unclear by multiplex PCR were further tested by either a combination of real-time PCR [11] and Quellung (for 2015 isolates) or by Quellung reaction alone (for 2017 isolates).

### Data Analyses

#### Definition of Vaccine and Nonvaccine Serotypes

The following serotypes, included in PCV13, were considered VTs: 1, 3, 4, 5, 6A, 6B, 7F, 9V, 14, 18C, 19A, 19F, and 23F. All other serotypes were categorized as nonvaccine types (NVTs).

#### Definition of a Valid Vaccination

Based on the PCV13 schedule in Burkina Faso, a valid dose was defined as a dose given at ≥8 weeks of age and ≥2 weeks before specimen collection. No intradose spacing requirement was used; of note, all subjects but 1 (in 2017) had ≥4 weeks between doses.

#### Statistical Analyses

We used χ ^2^ or Fisher exact tests to compare proportions and t tests to compare means. Analyses were stratified by age group and vaccination status and accounted for the cluster sampling design. For the comparisons of data across all 3 surveys, we extracted 2008 (pre-PCV) overall and individual serotype carriage rates in specific age groups from the published results [9]. Vaccine impact was assessed by comparing the proportions of VT carriage among colonized individuals by age group between the pre-PCV survey (2008) and each of the post-PCV surveys (2015 and 2017). Differences were considered statistically significant at *P* < .05 . All analyses were performed with Stata 13 software (StataCorp).

#### Ethical Approval and Consent to Participate

The 2 study protocols (2015 and 2017) were approved by the Ethics Committee for Health Research of Burkina Faso. In addition, the 2015 study, a collaboration between the Burkina Faso Ministère de la Santé and Agence de Médecine Préventive, was also reviewed and approved by the Commission Nationale de l’Informatique et des Libertés, France. The 2017 carriage study, a collaboration between the Burkina Faso Ministère de la Santé and the Centers for Disease Control and Prevention (CDC), was reviewed and approved by CDC’s institutional human subjects review board. We systematically sought written informed consents from participants before questionnaire administration and sample collection.

## RESULTS

### Characteristics of Survey Participants

A total of 992 (8 participants had no laboratory results) and 1005 participants were enrolled and included in the analyses in 2015 and 2017, respectively. In 2015 and 2017, respectively, 45.5% and 44.4% of participants were male. Few adults aged ≥18 years were smokers (6.7% [8 of 119] in 2015 and 2.8% [5 of 182] in 2017); the proportions of persons who reported using antibiotics in the 2 weeks before the surveys were 5.0% and 11.2%, respectively. Coal was the fuel most frequently used in cooking (60.6% in 2015 and 83.1% in 2017) (Supplementary Table 1). For the 2008 survey, available demographic information was limited to the published data [9]^.^ The 2008 study recruited participants up to age 39 years; the proportion of smokers among participants aged >14 years was 8.4%, and 43.7% of participants were male. By comparison, 45.4% and 45.2% of participants under age 40 years were male in 2015 and 2017, respectively.

### PCV13 Eligibility and Coverage by Age Group

In 2015, a total of 321 participants, representing 32.4% of all participants and 53.8% of those <5 years of age, were eligible for at least ≥1 PCV dose. In 2017, these numbers were 511, 50.9%, and 84.6%, respectively. Among children aged <12 months, 68.2% (2015) and 71% (2017) had received ≥2 doses of PCV. Among children aged 12–23 months, the proportions with ≥2 doses of PCV were 62.4% and 97.9% in 2015 and 2017, respectively. Among all children aged <5 years, these proportions were 42.8% and 74.0%, respectively (Supplementary Table 2). The PCV status of some participants could not be ascertained because of the absence of written proof of vaccination, particularly among those aged 12–23 months; in this group, 6.1% and 26.6% of participants had unknown PCV status in 2015 and 2017, respectively.

### Prevalence of Pneumococcal Carriage in 2015 and 2017

The prevalence of pneumococcal carriage among all study participants was 33.8% (95% confidence interval [CI], 30.8%–36.9%) in 2015 and 60.6% (57.4%–63.7%) in 2017, an increase likely due to different methods for pneumococcal detection for the 2 surveys (see Methods). Among children aged <12 months, pneumococcal carriage prevalences were 40.0% (95% CI, 33.4%–47.0%) in 2015 and 64.2% (57.2%–70.6%) in 2017 (Table 1), while VT pneumococcal carriage prevalences were 16.0% (11.6%–21.7%) (2015) and 23.9% (19.5%–30.3%) (2017). Among children aged 1–4 years, pneumococcal carriage prevalences were 50.1% (95% CI, 45.0%–55.3%) in 2015 and 68.2% (63.6%–72.4%) in 2017; VT pneumococcal carriage prevalences were 21.6% (17.9%–25.8%) (2015) and 21.6% (17.8%–26.1%) (2017). The prevalences of VT carriage among all participants were 13.7% (95% CI, 11.7%–16.0%) and 21.6% (18.9%–24.5%) in 2015 and 2017, respectively, representing 40.6% and 35.6% of all pneumococcal colonization (Supplementary Table 3). Among infants aged <12 months who were colonized with *S. pneumoniae*, the percentage with a VT pneumococcus was lower in children who received 3 PCV doses than in those who received no doses in 2015 (37.7% vs 100%; *P* = .03), but not in 2017 (35.3% vs 40.0%; *P* = .54) (Table 2).

**Table 1.** Overall and Vaccine-Type Pneumococcal Carriage Among All Study Participants, by Age Group and Survey Year, in Bobo-Dioulasso^a^

Age Group (No. in 2015/2017)	Participants With Pneumococcal Carriage % (95% CI)			
	All Carriage		VT Carriage	
	2015	2017	2015	2017
<1 y (200/201)	40.0 (33.4–47.0)	64.2 (57.2–70.6)	16.0 (11.6–21.7)	23.9 (19.5–30.3)
1 y (198/199)	53.0 (46.0–60.0)	73.4 (66.8–79.0)	19.7 (14.7–25.3)	20.6 (15.5–26.9)
2–4 y (199/204)	47.2 (40.3–54.3)	63.2 (56.3–69.6)	23.1 (17.7–29.6)	22.6 (17.4–28.7)
5–14 y (198/201)	20.7 (15.5–27.1)	65.2 (58.2–71.5)	7.6 (4.6–12.2)	28.9 (23.2–35.3)
≥15 y (197/200)	7.6 (4.6–12.3)	37.0 (30.5–44.0)	2.0 (0.8–5.3)	12.0 (8.2–17.2)
All ages (992/1005)	33.8 (30.8–36.9)	60.6 (57.4–63.7)	13.7 (11.7–16.0)	21.6 (18.9–24.5)

Abbreviations: CI, confidence interval; VT, vaccine-type.

^a^The 2015 and 2017 studies used different laboratory methods to detect carriage. Of 657 culture-negative samples from 2015 that were analyzed through *lyt*A, 85 (12.9%) were *lyt*A positive; repeated culture of these samples yielded 27 additional *Streptococcus pneumoniae* isolates.

**Table 2.** Vaccine-Type Pneumococci Among Pneumococcal Carriers Aged <5 Years by Vaccination Status and Age Group in Bobo Dioulasso, 2015 and 2017^a^

Age Group	2015				2017			
	No. With VT Carriage/Total (%)			*P* Value (3 vs 0 Doses)	No. With VT Carriage/Total (%)			*P* Value (3 vs 0 Doses)
	0 Doses	1 or 2 Doses	3 Doses		0 Doses	1 or 2 Doses	3 Doses	
<1 y	3/3 (100)	6/19 (31.6)	20/53 (37.7)	.03	2/5 (40)	15/31 (48.4)	24/44 (35.3)	.54
1 y	13/21 (61.9)	3/13 (23.1)	20/65 (30.8)	.01	0	1/6 (16.7)	30/98 (30.6)	NA
2–4 y	46/94 (48.9)	0	0	NA	20/42 (47.6)	3/8 (37.5)	17/54 (31.5)	.11

Abbreviations: NA, Not applicable; VT, vaccine-type.

^a^The 2015 and 2017 studies used different laboratory methods to detect carriage.

### Changes in VT Pneumococcal Carriage Among Pneumococcal Carriers After PCV13 Introduction

**Figure 1.**
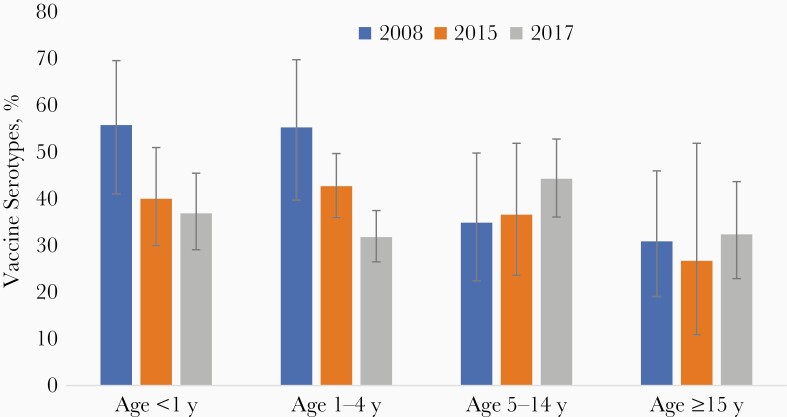
Proportions of vaccine-type carriage among all pneumococcal carriers by age group before and after 13-valent pneumococcal conjugate vaccine introduction in Bobo-Dioulasso, Burkina Faso. Error bars represent 95% confidence intervals.

Given that different laboratory methods were used to detect pneumococcal carriage among the 3 surveys, we analyzed the proportion of VT carriage among all pneumococcal carriers, by age group and study year (2008, 2015, or 2017) to evaluate the impact of PCV13 (Figure 1). Among infants aged <12 months, the percentage of pneumococcal carriers with a VT declined from 55.8% in 2008 (before PCV13 implementation) to 40.0% in 2015 (−28%) and 36.9% in 2017 (−34%) (difference between 2008 and 2017, 18.9%; 95% CI, 1.9%–35.9%; *P* = .03). Among children aged 1–4 years, the percentage of pneumococcal carriers with a VT declined from 55.3% in 2008 to 42.7% in 2015 (−23%) and 31.8% in 2017 (−42%) (difference between 2008 and 2017, 23.5%; 95% CI, 6.8%–40.2%; *P* = .004). There was no significant difference between surveys in the percentage of pneumococcal carriers with a VT in any of the other age groups, or in the survey population overall (Figure 1 and Supplementary Table 3).

### Serotype Distributions

Serotype data from each post-PCV13 carriage survey were analyzed separately. More than 1 serotype was identified among 32 (3.2%) participants in 2015 and 14 (1.4%) participants in 2017. Among children aged <5 years, the 3 VT serotypes with the highest carriage prevalence in 2015 were 19F (4%), 6A (3.9%), and 23F (2.8%); in 2017, these were 19F (5.8%), 23F (5.3%), and 3 (3.3%) (Table 3). In these children, the proportions of carriers colonized with serotypes 23F, 6A, 14, 19A, and 4 declined between 2008 and 2017, whereas those with serotypes 19F and 3 were fairly stable (Supplementary Figure 1). For participants ≥5 years of age, the proportions of carriers colonized with serotypes 23F, 6B, 18C, and 9V declined between 2008 and 2017, whereas those with serotypes 19F, 3, and 4 increased (Supplementary Figure 2).

**Table 3.** Distribution of Vaccine Serotype Pneumococci Among Children Aged <5 Years in Bobo-Dioulasso, 2015 and 2017^a^

Vaccine Serotype	Children, No. (%)	
	2015 (n = 597)	2017 (n = 604)
1	2 (0.3)	1 (0.2)
3	8 (1.3)	20 (3.3)
4	2 (0.3)	2 (0.3)
5	2 (0.3)	0 (0.0)
6A	23 (3.9)	13 (2.2)
6B	15 (2.5)	9 (1.5)
7F	1 (0.2)	0 (0.0)
9V	5 (0.8)	3 (0.5)
14	16 (2.7)	7 (1.2)
18C	2 (0.3)	7 (1.2)
19A	5 (0.8)	6 (1.0)
19F	24 (4.0)	35 (5.8)
23F	17 (2.8)	32 (5.3)
**Total**	**122 (20.4)**	**135 (22.4)**

^a^In 2015 and 2017, respectively, 318 (53.3%) and 200 (33.1%) specimens were pneumococcal culture negative among children aged <5 years; the numbers of nonvaccine serotypes in 2015 and 2017 were 163 (52.8%) and 244 60.4%), respectively. Note that laboratory methods used to detect carriage differed between 2015 and 2017.

## DISCUSSION

During 2017, approximately 3 years following introduction of the PCV13 into the national infant immunization program, we document a significant decline in the proportion of pneumococcal carriage due to PCV13 serotypes among children aged <1 year and 1–4 years compared with the pre-PCV period, with decreases of 34% and 42%, respectively. VT pneumococcal carriage persisted, however; approximately 20% of all participants still harbored VT pneumococci, as did about one-third of all children aged <1 year (37%) or 1–4 years (32%). Among participants ≥5 years of age, the proportion of pneumococcal carriers with a VT did not change significantly between 2008 and 2017, indicating that the infant PCV13 program had not stopped transmission of VT pneumococci among and to older children and adults, despite high national vaccine coverage, with 91% of children aged <12 months receiving 3 doses of PCV13 between 2014 and 2017 [13].

Overall and VT carriage prevalences were similar during 2008 [9] and 2015 but were higher in 2017 than in 2015. Because the 2017 laboratory procedures included a broth enrichment step shown to improve pneumococcal recovery [14, 15], the increase in overall pneumococcal carriage prevalence observed in 2017, 3 years after PCV introduction, was not unexpected. Nevertheless, when we evaluated the serotype distribution among pneumococci recovered, regardless of laboratory procedures used for overall detection of pneumococci (by estimating VT carriage among pneumococcal colonized individuals), our results were consistent with the literature. Indeed, as PCV programs mature and vaccine coverage increases, many studies that used consistent methods over time have shown consistent declines in VT serotypes [5, 16–20].

Unlike the crude comparisons, VT carriage adjusted for pneumococcal detection rates declined between studies (2008, 2015, and 2017) among the partially vaccine-eligible group of participants aged ≤5 years. These reductions likely reflect the combined direct and indirect effects of PCV13 and add to the evidence on the effectiveness of PCVs in reducing VT carriage among children [5, 18, 21–25].

The declines in the percentage of pneumococcal carriers with a VT among children aged <5 years in our study are more modest than the 74% decline reported for the same age group in Kilifi, Kenya, after 5 years of implementation of a 10-valent PVC (PCV10) program that included a catch-up campaign targeting children <5 years old [5]. Unlike our study’s finding, the authors also reported significant reductions among unvaccinated individuals in Kenya aged ≥5 years, demonstrating evidence of indirect protection among those too old to have been vaccinated. The indirect effect of PCV13 against VT carriage was also reported 3 years after implementation of a routine schedule in Malawi (including a catchup campaign targeting children <1 year old), with a 49% reduction in the percentage of pneumococcal carriers with a VT among mothers negative for human immunodeficiency virus [20]. The higher levels of reduction for Kenya and Malawi compared to Burkina Faso may reflect the benefit of conducting a catch-up campaign among children through age 5 years at the time of PCV introduction, a strategy that was not used in Burkina Faso. 

Besides these observational studies, the value of catch-up campaigns has also been shown by mathematical modeling. Data from Vietnam [26] suggested that a catch-up campaign among children aged ≤5 years implemented at the time of PCV introduction would be very effective, leading to a further 38% reduction of invasive pneumococcal disease above that seen by routine vaccination alone, and to a near-eradication of VT carriage in the same age group. Likewise, using data from the Kilifi research platform in Kenya [27], Flasche et al [28] showed that a catch-up campaign among children aged ≤5 years would lead to the prevention of 42% more invasive pneumococcal disease cases compared with vaccinating only age-eligible cohorts within 10 years of PCV program implementation. In its position paper recommending the introduction of PCVs in routine immunization programs, the Strategic Advisory Group of Experts on Immunization, World Health Organization, stated that “Maximized protection at the time of introduction of PCV10 or PCV13 can be achieved by providing 2 catch-up doses at an interval of at least 2 months to unvaccinated children aged 12–24 months and to children aged 2–5 years who are at risk of pneumococcal infection” [29].

Our carriage study findings provide some context for early results on the impact of PCV13 against pneumococcal meningitis in Burkina Faso. Two years after introduction, the incidence of pneumococcal meningitis had declined significantly by 76% among children aged <1 year and by 58% among those aged 1–4 years; however, reductions among children aged 5–14 years and adults were not statistically significant [8]. Both the early carriage and the meningitis data suggest that, in contrast to effects reported from higher-income countries [30–32], vaccine serotypes continue to circulate in vaccinated and unvaccinated age groups 3 years after PCV13 introduction; this suggests that more time or alternative vaccination strategies might be needed for the full effect of the infant program to be observed in Burkina Faso.

Our data suggest little to no vaccine effect on vaccine serotypes 3 and 19F carriage in all participants. These observations are similar to those reported by in the Gambia 5 years after PCV introduction [33]. In Kilifi, Kenya, however, Hammitt et al [5] reported substantial reductions in carriage of all vaccine serotypes, possibly owing to the catch-up campaign at introduction. We also observed a consistent increase in the proportion of the nonvaccine serotype 35B following vaccine introduction in all participants, confirming similar findings in Kenya [5] and the Gambia [33]. However, pneumococcal meningitis caused by serotype 35B after vaccine introduction in Burkina Faso did not show an increase comparable to that of the carriage of this serotype [34].

Burkina Faso lies entirely within the African meningitis belt, which has a unique pneumococcal epidemiology, specifically high incidence of serotype 1 disease after age 2 years, as documented before [35] and after [34] PCV introduction. In neighboring Ghana, where PCV13 was introduced in 2012 on the same schedule as used in Burkina Faso (3 + 0 schedule, no catchup campaign), a serotype 1–dominated pneumococcal meningitis outbreak was recorded in 2015–2016 with many PCV-ineligible cases, suggesting little herd protection, if any at all, from Ghana’s infant PCV13 program [36]. An infant PCV program, at least based on a 3 + 0 schedule, appears unable to control transmission of serotype 1 in meningitis belt countries [37].

In a phase IV immunogenicity trial comparing a “2 + 1” schedule (at age 6 weeks, 14 weeks, and 9 months) with the standard 3 + 0 schedule among infants in Burkina Faso, the booster dose elicited a robust memory response among its recipients; for instance, 99% of infants in the 2 + 1 schedule arm versus 75% of those in the 3 + 0 schedule had achieved putatively protective immunoglobulin G levels of 0.35 µg/mL against serotype 1 at 10 months of age [38]. These findings suggest that adding a booster dose to the infant schedule, as was recently done in Australia [39, 40], could improve control of pneumococcal carriage in Burkina Faso. However, this is speculative and even a late first year of life booster may have little impact on serotype 1 transmission among older persons. 

Consistent with previous pneumococcal colonization studies [9, 41], carriage of serotype 1 in our study was very rare, contrasting with its frequent implication in pneumococcal meningitis. Consequently, it may be that unique schedules or approaches to immunization, such as a booster during later childhood or direct vaccination of a large segment of the population as was done for persons aged 1–29 years with serogroup A meningococcal conjugate vaccine [42, 43], are required to address serotype 1 in the meningitis belt. Indeed, while several years of implementation will be required to gather data on the effectiveness of shifting from the current 3 + 0 schedule to the 2 + 1 schedule (as already envisaged in Burkina Faso [44]), we have evidence from both empirical [5, 43] and modeling [26, 28] data that mass vaccination targeting a broader age group could rapidly lead to the control of pneumococcal carriage and disease. Eventually, irrespective of the dosing schedule, meningitis belt countries may need to implement at least one catch-up mass vaccination campaign to achieve maximal program impact. The financial feasibility and practical modalities of such campaigns have yet to be explored.

The following limitations should be considered in the interpretation of our findings. First, although all 3 studies have several common characteristics such as age-stratified probabilistic cluster sampling and sample collection during the dry season, there were some variations in laboratory procedures. Indeed, while direct plating was used in 2008 [9] (Quellung for serotype confirmation) and 2015 (real-time PCR and Quellung for serotype confirmation), broth enrichment of specimens preceded plating in 2017 (Quellung for serotype confirmation), and this may explain the differences observed in the crude carriage rates between 2017 and 2008 or 2015. We attempted to minimize this potential bias by adjusting for pneumococcal recovery rate, which consisted of calculating and comparing the percent carriage of individual serotypes or groups of serotypes among pneumococcal carriers. In so doing, we found meaningful results consistent with time, PCV introduction and the literature. 

A second limitation was that all 3 studies were limited to sampling conducted in the urban setting of Bobo-Dioulasso, the second-largest city of Burkina Faso; thus, findings are not necessarily generalizable to the entire country, given that carriage often differs by setting, especially between urban and rural areas [41]. Third, the cross-sectional design added to the limited number of time points studied cannot fully account for temporal trends. Finally, as the vaccination status of some children could not be ascertained, this brings uncertainty to our estimation of vaccine impact.

In conclusion, 3 years after PCV13 introduction in Burkina Faso, we showed significant reductions in the percentage of pneumococcal carriers with a vaccine serotype among the partially vaccine-eligible group of children aged <5 years. However, we found no clear evidence of indirect effects (herd immunity) among children aged ≥5 years or adults. Furthermore, in 2017, 1 in 5 children <2 years of age still harbored VT pneumococci. The lack of a catch-up campaign at the time of PCV13 introduction, the lack of a booster dose in the current PCV13 schedule, conditions in the meningitis belt that foster transmission, or a combination of multiple factors could explain the suboptimal clearance of vaccine serotypes among vaccine-eligible children and the absence of indirect effects against VT colonization. These findings, along with meningitis data, support consideration of changes in the implementation of PCV13 programs in Burkina Faso and similar settings.

## Supplementary Data

Supplementary materials are available at The Journal of Infectious Diseases online. Consisting of data provided by the authors to benefit the reader, the posted materials are not copyedited and are the sole responsibility of the authors, so questions or comments should be addressed to the corresponding author.

jiab037_suppl_Supplementary_Material

## Notes

***Acknowledgments.*** The authors extend their sincere acknowledgements to Tahirou Ouro-Akpo and Oumar Sanou for their contribution as biologists, as well as to Armel Bambara for his contribution as data manager in 2015. They also thank Eli Kabré (Centre Muraz) and Theresa Tran and Hollis Walker (Centers for Disease Control and Prevention) for assistance with processing of swab samples and serotyping of isolates from 2017.

***Author contributions.*** Study conception and design: L. K., T. A., B. M. N. L., B. B., S. Y., E. B., B. D. G., C. G. W., J. C. M., and C.A.V.B. Survey data collection: L. K., T. A., F. T. T., F. A., S. Y., and C. A. V. B. Sample collection: T. A., B. M. N. L., B. M., and L. M. Laboratory analyses: B. M. N. L., S. O., S. V., and L. M. Data management and cleaning: L. K., T. A., F. T. T., and J. C. M. Data analysis: L. K., T. A., J. C. M., and C. A. V. B. Manuscript drafting: L. K., A. G., and J. C. M. Manuscript critical review: B. M. N. L., B. B., L. M., E. B., A. G., B. D .G., C. G. W., J. C. M., and C. A. V. B. All authors read and approved the final manuscript.

***Disclaimer.*** The findings and conclusions in this report are those of the authors and do not represent the official position of the US Centers for Disease Control and Prevention. The funding source had no role in the design of the study and collection, analysis, and interpretation of data and in writing the manuscript.

***Supplement sponsorship.*** This supplement is sponsored by the World Health Organization and the U. S. Centers for Disease Control and Prevention.

***Financial support.*** This work was supported by Gavi, the Vaccine Alliance.

***Potential conflicts of interest.*** J. C. M. and B. D. G. initiated the study while at the Agence de Médecine Préventive but are now employed by Pfizer. All other authors report no potential conflicts. All authors have submitted the ICMJE Form for Disclosure of Potential Conflicts of Interest. Conflicts that the editors consider relevant to the content of the manuscript have been disclosed.
