# Supplementary material for: Pneumococcal Carriage in Burkina Faso After 13-Valent Pneumococcal Conjugate Vaccine Introduction: Results From 2 Cross-sectional Population-Based Surveys
Source: J Infect Dis. 2021 Sep 1;224(Suppl 3):S258–66. doi: 10.1093/infdis/jiab037 (PMC8409529; doi:10.1093/infdis/jiab037)
Supplement: jiab037_suppl_Supplementary_Material [file jiab037_suppl_supplementary_material.docx]

**SUPPLEMENTAL MATERIAL**

Supp. Table 1. Demographic and epidemiological characteristics of participants enrolled in pneumococcal carriage surveys following PCV13 introduction—Burkina Faso, 2015 and 2017

| **Characteristic** | **Surveys** | |
| --- | --- | --- |
|  | **2015 (n=992)**  No. (%) | **2017 (n=1005)**  No. (%) |
| *Age* |  | |
| <1yr | 200 (20.2) | 201 (20.0) |
| 1yr | 198 (19.9) | 199 (19.8) |
| 2-4 years | 199 (20.1) | 204 (20.3) |
| 5-14 years | 198 (19.9) | 201 (20.0) |
| ≥15 years | 197 (19.9) | 200 (19.9) |
| *Male sex* | 451 (45.5) | 446 (44.4) |
| *Respiratory symptoms at time of enrollment* | 534 (53.9) | 487 (48.5) |
| *Crowding:* |  | |
| ≥6 persons in the household | 461 (46.5) | 574 (57.2) |
| ≥3 persons sharing a room | 683 (68.8) | 561 (56.0) |
| *Smoker (>= 18 years)* | 8/119 (6.7) | 5/182 (2.8) |
| *Use of antibiotics ≤ 2 weeks before specimen collection* | 50 (5.0) | 113(11.2) |
| *Type of fuel used for cooking* |  | |
| gas | 130 (13.1) | 312 (31.0) |
| coal | 601 (60.6) | 835 (83.1) |
| wood | 353 (35.6) | 488 (48.6) |
| *Possessions in the household:* |  | |
| radio | 671 (67.6) | 753 (74.9) |
| TV | 736 (74.2) | 830 (82.6) |
| phone | 977 (98.5) | 983 (97.8) |
| motorbike | 756 (76.2) | 810 (80.6) |

| **Number of PCV dose**s | **Age group and study year** | | | | | | | |
| --- | --- | --- | --- | --- | --- | --- | --- | --- |
|  | **< 1 year** | | **1 year** | | **2-4 years** | | **All children aged <5 years** | |
|  | 2015  n (%) | 2017  n (%) | 2015  n (%) | 2017  n (%) | 2015  n (%) | 2017  n (%) | 2015  n (%) | 2017  n (%) |
| 0 | 34 (17.7) | 20 (12.3) | 55 (29.6) | 0 (0) | 199 (100) | 71 (43.0) | 288 (49.9) | 91 (19.2) |
| 1 | 27 (14.1) | 27 (16.7) | 15 (8.0) | 3 (2.1) | 0 (0) | 2 (1.2) | 42 (7.3) | 32 (6.8) |
| 2 | 34 (17.7) | 19 (11.7) | 13 (7.0) | 5 (3.4) | 0 (0) | 8 (4.9) | 47 (8.1) | 32 (6.8) |
| 3 | 97 (50.5) | 96 (59.3) | 103 (55.4) | 138 (94.5) | 0 (0) | 84 (50.9) | 200 (34.7) | 318 (67.2) |

Supp. Table 2. Number of PCV doses by age group among children aged less than 5 years in 2015 and 2017, Bobo-Dioulasso.

Note. Some participants had unknown vaccination status, so the numbers in each column do not necessarily add up to the size of each group shown in Supp. Table 1.

| **Age group** | **2008** | | **2015** | | **2017** | | **p values** | | |
| --- | --- | --- | --- | --- | --- | --- | --- | --- | --- |
|  | **Any Sp N** | **VT Sp**  **n (%*)** | **Any Sp N** | **VT Sp**  **n (%*)** | **Any Sp N** | **VT Sp**  **n (%*)** | **2008 vs 2015** | **2008 vs 2017** | **2015 vs 2017** |
| < 6 months | 21 | 10  (47.6) | 22 | 11 (50.0) | 53 | 25  (47.2) | 0.88 | 0.97 | 0.82 |
| 6-11 months | 22 | 14  (63.6) | 58 | 21 (36.2) | 77 | 23  (29.9) | **0.03** | **0.004** | 0.44 |
| < 12 months | 43 | 24  (55.8) | 80 | 32 (40.0) | 130 | 48  (36.9) | 0.09 | **0.03** | 0.66 |
| 1-4 years | 38 | 21  (55.3) | 199 | 85 (42.7) | 274 | 87  (31.8) | 0.15 | **0.004** | **0.01** |
| 5-14 years | 43 | 15  (34.9) | 41 | 15 (36.6) | 131 | 58  (44.3) | 0.87 | 0.289 | 0.39 |
| ≥15 years | 42 | 13  (30.9) | 15 | 4 (26.7) | 74 | 24  (32.4) | 0.48 | 0.92 | 0.66 |
| Total | 166 | 73  (44.0) | 335 | 136 (40.6) | 609 | 217 (35.6) | 0.47 | **0.04** | 0.13 |

Supp. Table 3. Comparisons of vaccine-type carriage among pneumococcal (Sp) carriers by age group between study years, Bobo-Dioulasso.

*Percent of pneumococcal carriers that had a vaccine serotype

Supp. Figure 1. Serotype distribution of isolated pneumococci among children <5 years of age before and after PCV13 introduction, Bobo-Dioulasso, Burkina Faso


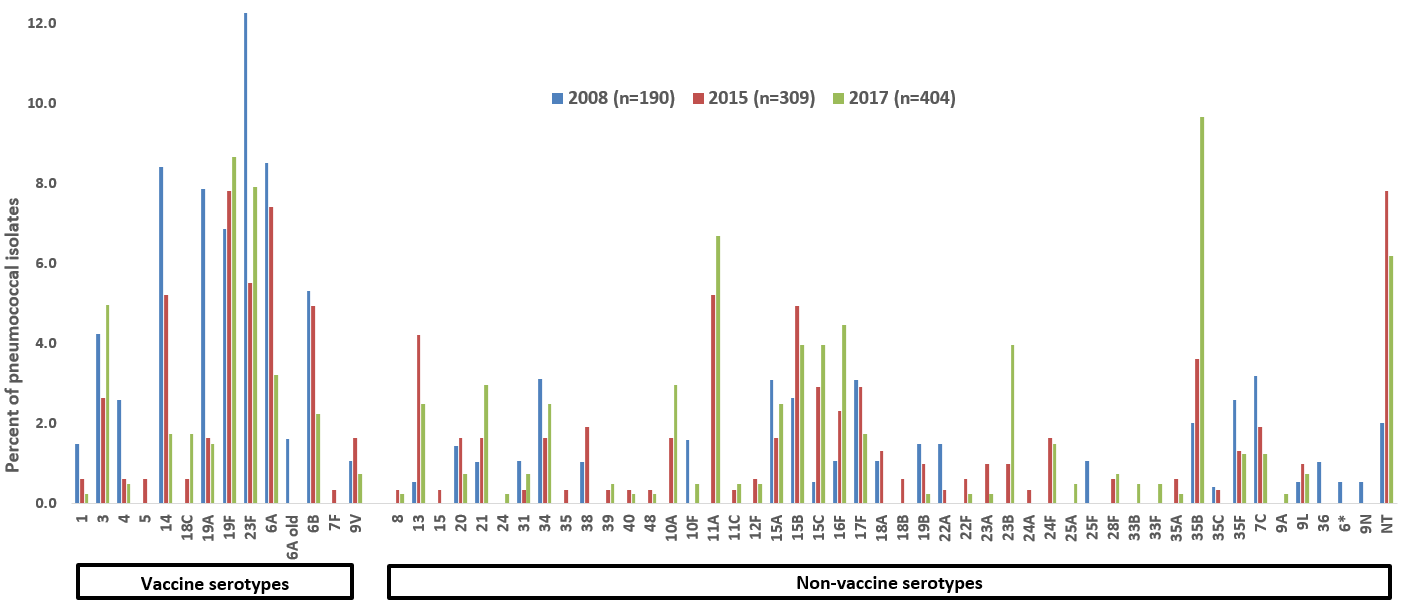


Note: Serotype results for the 2008 carriage study were based on the Quellung reaction; serotype results for serogroup 6 shown here (“6A”, “6B” and “6A old”) are taken from the original publication [9] and cannot be further characterized. In 2008, Quellung results for 6A included both 6A+6C (Quellung was not able to distinguish 6C from 6A in 2008 [9].

Supp. Figure 2. Serotype distribution among isolated pneumococci in participants aged ≥ 5 years before (2008) and after (2015 and 2017) PCV13 introduction, Bobo-Dioulasso.


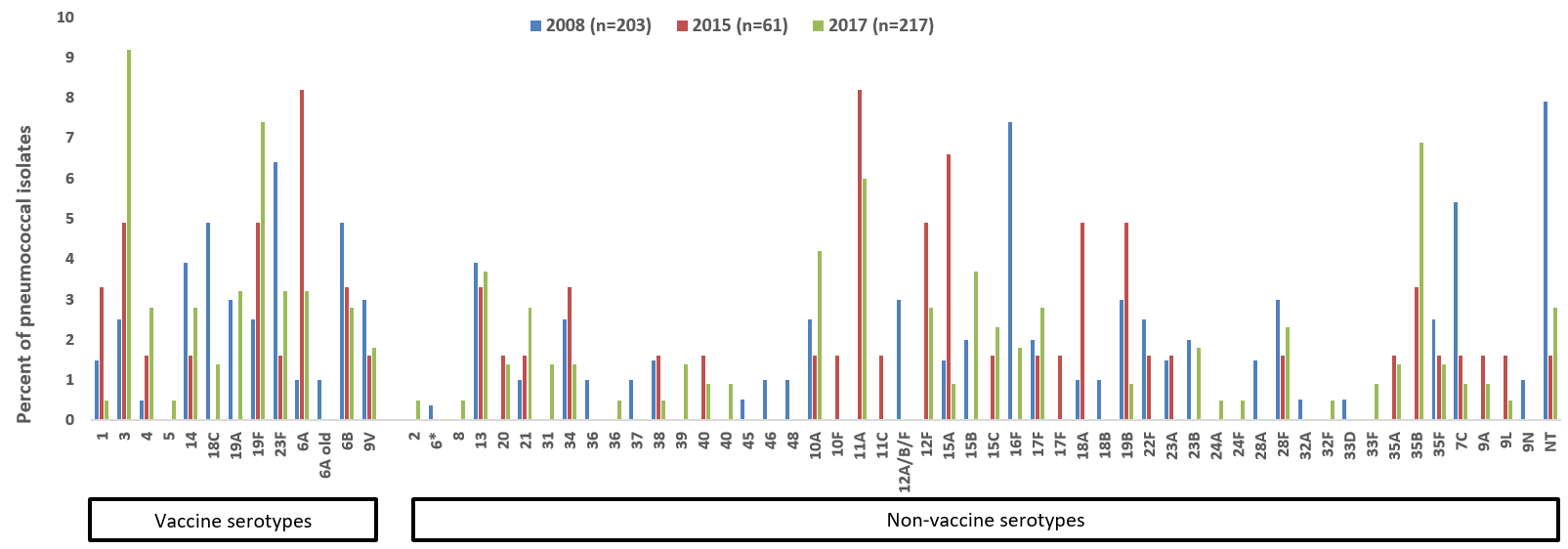


Note: Serotype results for the 2008 carriage study were based on the Quellung reaction; serotype results for serogroup 6 shown here (“6A”, “6B” and “6A old”) are taken from the original publication [9] and cannot be further characterized. In 2008, Quellung results for 6A included both 6A+6C (Quellung was not able to distinguish 6C from 6A in 2008 [9].
